# Transcriptome Analysis of the Anti-Proliferative Effects of Ginsenoside Rh3 on HCT116 Colorectal Cancer Cells

**DOI:** 10.3390/molecules27155002

**Published:** 2022-08-06

**Authors:** Siying Teng, Xi Lei, Xinmin Zhang, Dongming Shen, Qiuyi Liu, Yingjie Sun, Yi Wang, Zhongyi Cong

**Affiliations:** 1 Department of Regenerative Medicine, School of Pharmaceutical Science, Jilin University, Fujin Road 1266, Changchun 130021, China; 2 Department of Ophthalmology, The First Hospital of Jilin University, Jilin University, Xinmin Street 1, Changchun 130021, China

**Keywords:** ginsenoside (Rh3), colorectal cancer, transcriptome analysis, proliferation, DNA replication

## Abstract

The mechanism of ginsenoside Rh3 activity against cancer remains unclear. This study aimed to investigate the underlying mechanism. The effects of Rh3 on the cell proliferation, migration and invasion, and cycle and apoptosis were analyzed using CCK-8 assay, transwell migration assay and flow cytometry, respectively. The RNA transcriptome was sequenced and data were analyzed by R software. Protein expression and protein-protein interactions were determined by Western blotting and co-immunoprecipitation, respectively. The results showed Rh3 inhibited HCT116 cell proliferation, invasion, and migration, arrested cells at G1 phase; and increased apoptosis. Rh3 downregulated 314 genes and upregulated 371 genes. Gene Set Enrichment Analysis (GSEA) using *The Kyoto Encyclopedia of Genes Genomics* ranked DNA replication first, while GSEA using Gene Ontology ranked the initiation of DNA replication first. Compared with tumor data from The Cancer Genome Atlas (TCGA), most of genes related to DNA replication were oppositely regulated by Rh3. Furthermore, Rh3 down-regulated key protein expression related to DNA replication (Orc6, Cdt1, and Mcm2), but did not affect the loading of Mcm complexes onto ORC complexes nor the phosphorylation at ser139 of Mcm2. Therefore, Rh3 may inhibit colorectal cancer HCT116 cells by downregulation of genes related to DNA replication.

## 1. Introduction

Colorectal cancer is the third most commonly diagnosed cancer (10.0% of total cases), and the second cause of cancer death (9.4% of the total cancer deaths) worldwide. More than 1.9 million new colorectal cancer cases and 935,000 deaths were estimated to occur in 2020. The incidence rates are approximately four-fold higher in transitioned countries compared with transitioning countries, but there is less variation in the mortality rates [1]. In China, the estimated total new colorectal cancer cases and deaths in 2022 were 592,232 and 309,114 respectively, and incidence and mortality rates are increasing [2]. For patients with early stages of colorectal cancer, local surgical excision shows good prognosis. As the disease only becomes symptomatic at an advanced stage, some patients have missed the opportunity of surgery when they are diagnosed. For these patients, palliative chemotherapy is still the main treatment. Although new options, such as targeted therapy and immunotherapy, have improved the 3-year overall survival rate of patients, their wide application is limited by the choice of indications and high cost [3]. Chemotherapy can cause much damage, including toxicity to healthy cells, pain, fatigue, diarrhea, vomiting, hair loss, immunosuppression and reduction in bone density [4]. In addition, chemotherapy drugs cannot kill all cancer cells in a short time, and long-term application leads to acquired drug resistance. Therefore, it is necessary to find other ways to treat colorectal cancer with low toxicity.

Ginseng is a traditional Chinese health food and medicine. Ginsenosides are steroid glycosides and the main active components of ginseng. Previous in vitro and animal studies have reported that these compounds have anti-oxidation [5], anti-inflammation [6], and anti-tumor [7] effects, and might also improve memory [8]. There is increasing attention given to the anti-tumor activity of ginsenosides. The ginsenosides are divided into protopanaxadiols and protopanaxatriols based on the position of hydrophilic sugar moieties. Protopanaxadiols, such as Rg1, Rg2, Rg3, and Rh2, have much stronger anti-tumor activity than protopanaxatriols [9].

Rh3 is a rare but important protopanaxadiol, and many studies have reported it has a variety of pharmacological and physiological effects mediated by different mechanisms. Rh3 protected rat myocardial tissues against myocardial ischemia-reperfusion injury by inhibiting cell apoptosis via suppression of the p38 MAPK pathway [10]. It also protected murine endometrial cells from oxygen and glucose deprivation-reoxygenation by activating the Nrf2 signaling pathway via induction of the phosphorylation of Nrf2 Ser-40 and the dissociation of Keap1-Nrf2 [11]. Rh3 inhibited ultraviolet radiation-induced damage by inhibiting epidermal growth factor receptor (EGFR), protein kinase C, and extracellular signal-regulated kinase [12,13], and activating nuclear-factor-E2-related factor 2 signaling [14]. Rh3 can reduce the inflammation of microglia induced by lipopolysaccharide (LPS) via regulating the AMP pathway and its downstream signaling pathways [15]. It also reduced cisplatin-induced apoptotic damage in renal proximal LLC-PK1 cells by regulating JNK and ERK phosphorylation in the MAPK signaling pathway [16]. In addition, our previous study found that Rh3 inhibited the proliferation of colorectal cancer cells in a dose- and time-dependent manner, and induced cell apoptosis via upregulating the expression of caspase-3 [17].

In this study, we first examined the effect of Rh3 on in vitro cell viability, cycle progression, invasion, and migration of colorectal cancer HCT116 cells. We then used RNA-seq to examine the effects of Rh3 on the transcriptome profile to identify differentially expressed genes (DEGs), and used the *Kyoto Encyclopedia of Genes and Genomes* (KEGG) and Gene Ontology (GO) Gene Set Enrichment Analysis (GSEA) to interpret these gene expression data. Finally, the expression and interactions of the key genes of the first-ranked biological processes and pathways were further validated using Western blotting.

## 2. Results

### 2.1. Effect of Rh3 on Viability, Migration, Invasion, and Cell Cycle Progression in HCT116 Cells

**Figure 1:**
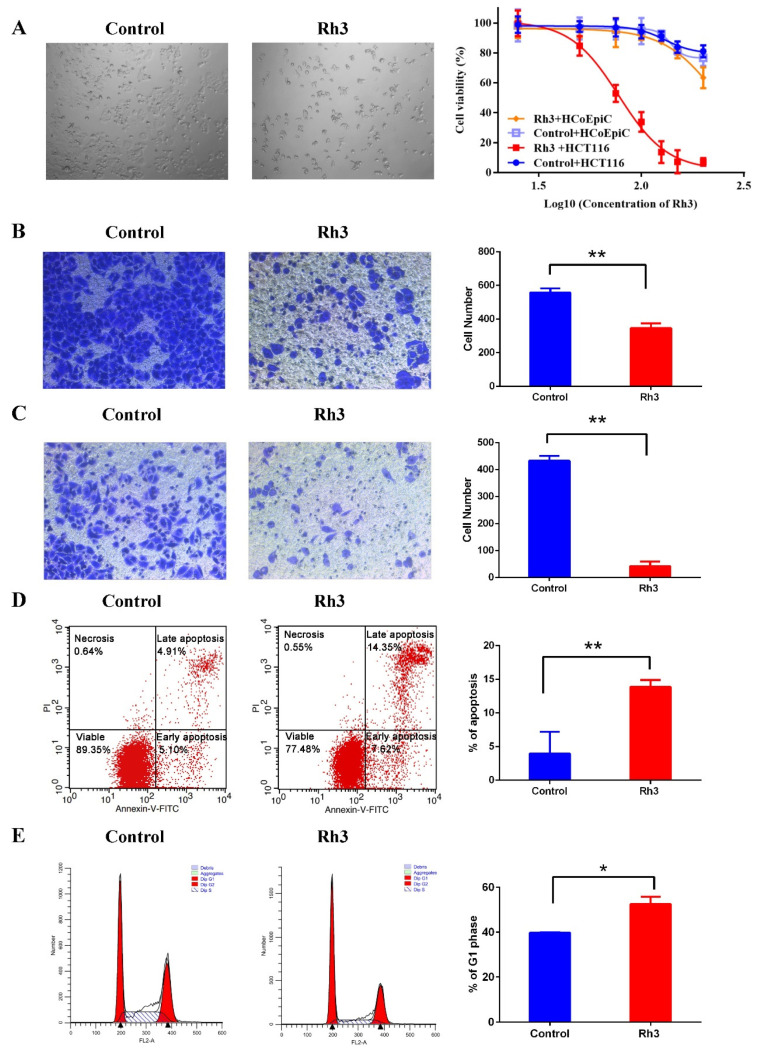
Effect of Rh3 on cell viability, migration, invasion, and cell cycle progression in HCT116 cells. (**A**) Cell proliferation after treatment with different concentration of Rh3 (25, 50, 75, 100, 125, 150 or 200 μg/mL) or the same amount of solvent (DMSO) for 24 h (CCK-8 assay, photo magnification 40×). (**B**,**C**) Effect of Rh3 on HCT116 cell migration and invasion (transwell assay, photo magnification 100×). (**D**,**E**) Apoptosis and cell cycle analyses after treatment with Rh3 for 24 h (flow cytometry). Groups were compared using a two-tailed Student’s *t*-test. Error bars: standard deviation, * *p* < 0.05, ** *p* < 0.01.

We first assessed the inhibitory effect of Rh3 on HCT116 cells using the CCK-8 assay. The results showed that the IC_50_ of Rh3 in colorectal cancer cells (HCT116) was 77.54 μg/mL, and in human normal colon epithelial cells (HCoEpiC) was 235.8 μg/mL (Figure 1A and Figure S1); thus, the SI_Rh3_ was 3.04. Therefore, we performed subsequent experiments using an Rh3 concentration of 80 μg/mL. Transwell assays indicated that Rh3 treatment greatly reduced cell migration and invasion relative to the DMSO control (Figure 1B,C). Flow cytometry assays demonstrated that Rh3 increased the proportion of apoptotic cells (Figure 1D) and the percentage of cells in the G0/G1 phase (Figure 1E). Taken together, these results reveal that Rh3 suppressed HCT116 cell proliferation, migration, and invasion, induced cell cycle arrest in the G0/G1 phase, and increased cell apoptosis.

### 2.2. Effect of Rh3 on Gene Transcription in HCT116 Cells

**Figure 2:**
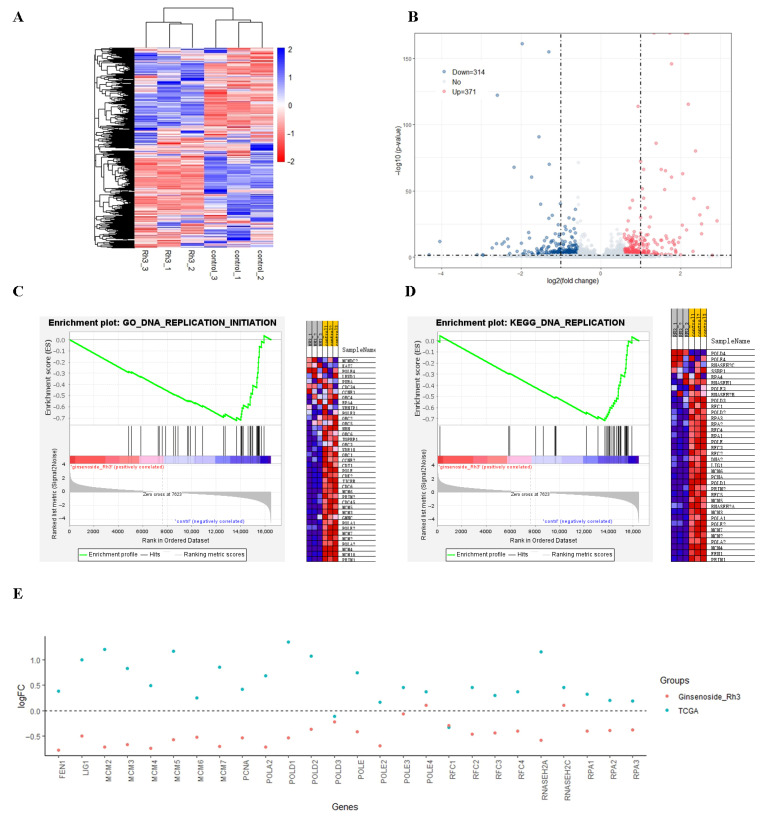
Effect of Rh3 on gene transcription in HCT116 cells. (**A**) Heatmap of genes with differential expression. Colored bars indicate scaled reads per kilobase of transcript (RPKM) of each gene (blue: high expression in the Rh3 group, red: low expression in the Rh3 group). Dendrograms represent similarities in clusters of samples (left) and in levels of gene expression (top). (**B**) Volcano plot of differential gene expression. The horizontal dashed line indicates *p* = 0.05, and the vertical dashed lines indicate log_2_FC = +1 and log_2_FC = −1. (**C**,**D**) GSEA of GO and KEGG pathways with differential gene expression (GSEA version 4.1.0). The first ranks of GSEA of the GO and KEGG pathways are DNA replication initiation and DNA replication pathways; other pathways are shown in Tables S1 and S2. (**E**) Log_2_FC in the expression of specific genes that function in DNA replication in colon cancer tumor tissue relative to adjacent normal colon tissue (blue, from TCGA), and in the Rh3 group relative to the DMSO group (red).

To verify the RNA sequencing results, we randomly selected 11 differentially expressed genes followed by real-time quantitative PCR (qPCR). The qPCR results showed that most of the changes were consistent with the results from high-throughput sequencing (Figure S2). Our heatmap analysis of the effect of Rh3 on gene transcription indicated clustering into distinct groups (the raw data of RNA-seq were submitted to the Sequence Read Archive of National Center for Biotechnology Information. BioProject ID PRJNA765451). In particular, the gene expression patterns of the Rh3 and control groups were different, and gene expression of samples in the same group had high similarity (Figure 2A). A comparison of transcriptome profiles of the Rh3 and control groups demonstrated there were 685 DEGs (|log_2_FC| > 0.585 and false discovery rate [FDR] < 0.05), with 314 down-regulated and 371 up-regulated genes in the Rh3 group (Figure 2B). GSEA (based on GO and the KEGG) showed that the initiation of DNA replication and the DNA replication pathway ranked first (Figure 2C,D; Tables S1 and S2).

To verify the potential of Rh3 as an anti-colon cancer drug, we downloaded data on colon cancer gene expression and associated clinical variables of these patients from the TCGA database, with a focus on genes related to DNA replication. A comparison indicated that cancer tissues had greater expression of most of these genes relative to adjacent normal tissues (blue dots in Figure 2E). Notably, Rh3-treated cells had lower expression of most of these same genes relative to DMSO-treated cells (red dots in Figure 2E).

### 2.3. Effect of Rh3 on Expression of Proteins Related to the Initiation of DNA Replication

**Figure 3:**
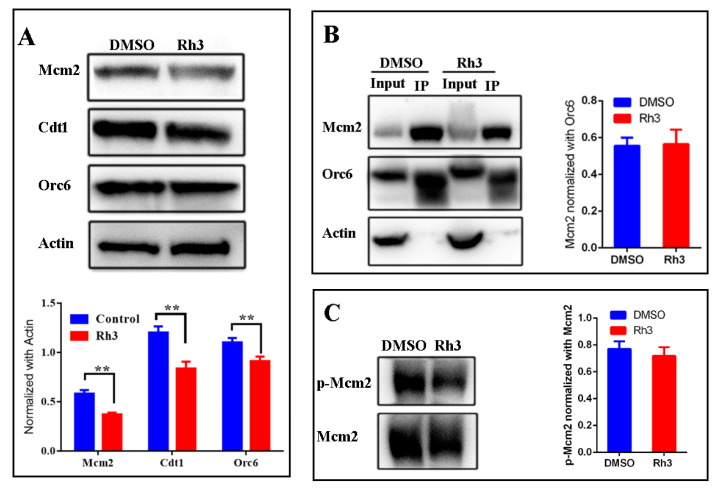
Effect of Rh3 on the expression of DNA replication initiation proteins. (**A**) Expression of Mcm2, Cdt1, and Orc6 in HCT116 cells (Western blotting with actin as the loading control). (**B**) Effect of Rh3 on the interaction between Orc6 and Mcm2 (Co-IP assay). (**C**) Expression of Mcm2 and phosphorylated Mcm2 (western blotting). Groups were compared using a two-tailed Student’s *t*-test. ** *p* < 0.01.

Our flow cytometry results indicated that Rh3-induced cell cycle arrest at G1 phase, during which cells normally synthesize multiple enzymes and nutrients needed for DNA replication. Therefore, we used Western blotting to examine the expression of three key proteins related to the initiation of DNA replication: Orc6, Cdt1, and Mcm2. The results show that all three proteins were down-regulated in the Rh3 group relative to the control group (Figure 3A).

During the initiation of eukaryotic DNA replication in G1 phase, the six-subunit origin recognition complex (ORC) binds to the replication origins [18], Cdt1 is tethered to Orc6 during the loading of the mini-chromosome maintenance 2–7 (Mcm2-7) helicase, and this forms an ORC-Cdc6-Cdt1-Mcm2-7 (OCCM) helicase-loading intermediate [19,20,21]. The phosphorylation of Mcm2 is a sign of the beginning of S phase and DNA synthesis [22]. Analysis of the effect of Rh3 on the formation of the OCCM helicase-loading intermediate using a co-immunoprecipitation (Co-IP) assay showed that complex formation was not significantly different between the control and Rh3 groups (Figure 3B). Western blotting indicated no significant difference in the phosphorylation level of Mcm2 between the control and Rh3 groups (Figure 3C). These results demonstrated that although Rh3 down-regulated the expression of the Orc6, Cdt1, and Mcm2 proteins, it did not affect the loading of MCM complexes on ORC complexes nor the phosphorylation at ser139 of Mcm2.

### 2.4. Effect of Rh3 on Expression of Proteins Related to Cell Migration and Invasion

**Figure 4:**
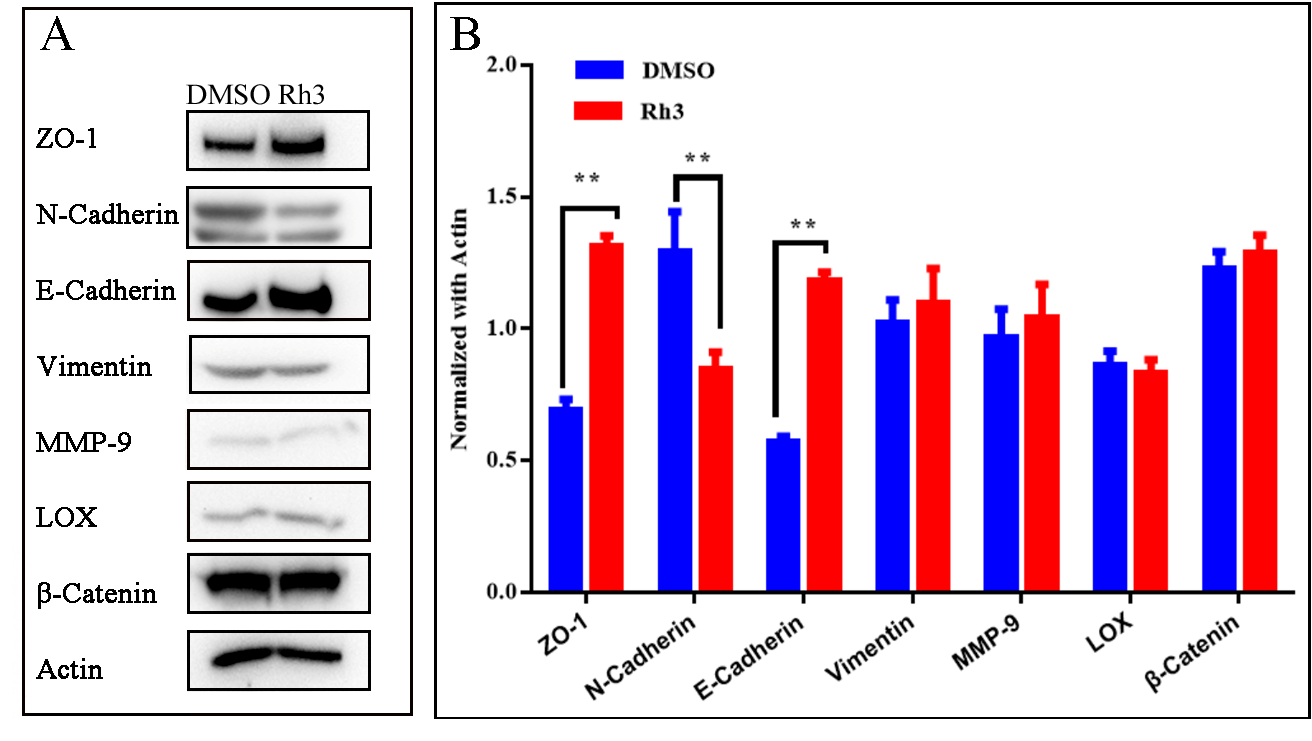
Effect of Rh3 on the expression of invasion and migration associated proteins. **(A):** Expression of ZO-1, N-Cadherin, E-Cadherin, Vimentin, MMP-9, β-Catenin, and LOX in HCT116 cells were detected by Western blotting with actin as the loading control. (**B**) Gray analysis of western blotting on ZO-1, N-Cadherin, E-Cadherin, Vimentin, MMP-9, LOX, and β-Catenin. Groups were compared using a two-tailed Student’s *t*-test. ** *p* < 0.01.

The results of transwell assays indicated that Rh3 treatment greatly reduced cell migration and invasion relative to the DMSO control (Figure 1B,C). Since apoptosis and necrosis induced by Rh3 might influence the results of the transwell assays, we further analyzed the expression of proteins that promote cell invasion and migration (N-Cadherin, Vimentin, matrix metalloproteinase-9 and lysyl oxidase) and proteins that inhibit invasion and migration (zonula occludens-1, β-Catenin, and E-Cadherin). The results show that Rh3 had no effect on the expression of Vimentin, matrix metalloproteinase-9 (MMP-9), β-Catenin, or lysyl oxidase (LOX), but it up-regulated the expression of E-Cadherin and zonula occludens-1 (ZO-1), and down-regulated the expression of N-Cadherin (Figure 4).

## 3. Discussion

The results of this study demonstrated Rh3 inhibited the proliferation, migration, and invasion of HCT116 cells, arrested these cells in the G1 phase, and increased cell apoptosis. Rh3 is a rare ginsenoside whose chemical structure is similar to that of Rh2. Both of these compounds are tetracyclic triterpenoids, and they differ only at Carbon-20, where Rh3 has a diene bond and Rh2 has a carbon-carbon single bond and a hydroxyl group. Many studies have examined the effects of Rh2 on different tumor cells and documented that it inhibited cell proliferation, induced apoptosis, and arrested the cell cycle [23,24,25,26]. Other tetracyclic triterpenoids, such as ginsenoside Rg3 [27], Rg5 [28] and compound K [29], can also inhibit tumor cell proliferation. These results suggested that the tetracyclic triterpene ring of these compounds may be responsible for their anti-tumor function. However, our previous hemolysis experiments showed that Rh2 and Rg3, which are structurally similar to Rh3, had strong hemolytic effects, while Rh3 didn’t have this disadvantage [17].

Our group and other groups previously described the mechanism underlying the diverse biological and pharmacological activities of Rh3 by analysis of a few specific genes and pathways that were related to apoptosis [16,17], inflammation [30], oxidative stress [11,14], and other biological processes [31]. However, these previous analyses potentially neglected many other genes that were also regulated by Rh3. In the present study, we analyzed the effect of Rh3 on the entire transcriptome of colorectal cancer cells (HCT116) using RNA-seq. Our results indicated that Rh3 down-regulated 314 genes and up-regulated 371 genes. Because we only selected DEGs during the enrichment analysis, and expression patterns are unbiased in the GSEA [32], we used GSEA to analyze the key biological processes affected by Rh3. Previous research reported that Rh3 affected several important pathways and biological processes, such as MAPK [10], apoptosis [16], and the ERK1 and ERK2 cascade [12]. The present study is the first to demonstrate that Rh3 also altered pathways closely related to the cell cycle, development, differentiation, the immune network, and the Hedgehog and calcium signaling pathway. Importantly, we found that DNA replication initiation in the GO GSEA and the DNA replication pathway in the KEGG GSEA ranked first (Tables S1 and S2). Furthermore, the same genes related to initiation of DNA replication that were down-regulated by Rh3 in HCT116 cells were up-regulated in tumor tissues from TCGA (Figure 2E). Interestingly, some established chemotherapeutic agents, such as 5-fluorouracil [33], exert potent anti-tumor activity by inhibiting DNA replication.

The molecular mechanism by which cells regulate DNA replication is very complicated. During the G1-phase of the cell cycle, the essential replication factors, ORC complex, Cdc6, Cdt1, and Mcm2-7, assemble at replication origins to establish pre-replicative complexes [34,35]. Our flow cytometry analyses showed that Rh3 treatment of HCT116 cells led to cell cycle arrest in the G1 phase. In addition, the Western blotting results indicated that Rh3 down regulated key DNA replication initiation proteins (Orc6, Cdt1, and Mcm2), but did not affect the loading of MCM complexes on ORC complexes nor the phosphorylation of Mcm2. It is possible there were excessive DNA replication-related proteins in these cells, and the phosphorylases (Dbf4-dependent kinases) of Mcm2 might not be excessive; thus, Rh3 had no obvious effect on the phosphorylation level of Mcm2. Therefore, we suggest that Rh3 might reduce the proliferation of colorectal cancer cells (HCT116) by altering the expression of proteins involved in the initiation of DNA replication, but not modify these proteins after expression.

The extracellular matrix (ECM) is a three-dimensional network structure composed of two major classes of biomolecules: glycosaminoglycans (which are linked to proteins to form proteoglycans), and fibrous proteins (including collagen, elastin, and fibronectin). The ECM provides nutrition, physical support, and functions as a barrier, but also regulates tumor cell invasion and migration [36]. In addition, during metastasis, tumor cells undergo morphological changes, lose their polarity, and have decreased inter-cellular interactions, a process known as epithelial-to-mesenchymal transition (EMT) [37]. LOX can oxidize collagen and elastin to form covalent cross-links and create a stable network structure. This structure provides non-specific resistance to proteolysis, thereby inhibiting tumor metastasis; however, in a hypoxic environment, high expression LOX creates an ECM that is excessively cross-linked and rigid, and this promotes tumor cell invasion and metastasis [38]. MMPs can degrade various protein components in the ECM and destroy this functional barrier to cell invasion. The main function of MMP-9 is degradation of collagens, gelatin, elastin, proteoglycan, fibronectin, and related proteins. MMP-9 was found to be overexpressed in a variety of tumors [39], and its expression is positively correlated with tumor cell invasion and migration [40]. β-Catenin is a component of cell-cell adhesion, and mainly mediates tumor cell migration via the wnt pathway [41]. Cadherins are Ca^2+^ dependent cell surface molecules involved in cell adhesion and morphogenetic and differentiation processes during development. Inappropriate N-Cadherin expression has an invasion-promoting effect on colon cancer [42], and up-regulation of N-Cadherin followed by the downregulation of E-Cadherin is the hallmark of EMT [37]. ZO-1 forms a continuous liquid barrier between cells, maintains cell polarity by regulating cell permeability, and regulates the binding of transmembrane proteins to the cellular skeleton [43]. Vimentin is a conserved intermediate filament family protein, is widely expressed in normal mesenchymal cells, and is known to maintain cellular integrity and provide stress resistance. Along with Cadherin, ZO-1 and Vimentin are well-characterized markers of the EMT. Decreased expression of E-Cadherin and ZO-1, and increased expression of N-Cadherin and Vimentin have been correlated with tumor metastasis. To more accurately analyze the effects of Rh3 on cell migration and invasion, and exclude the influence of apoptosis and necrosis, we performed experiments using transwell assays and Western blotting. The transwell experiments indicated that Rh3 affected cell migration and invasion. The Western blotting experiments indicated that Rh3 did not affect the expression of matrix degradation related enzymes (LOX and MMP) or the expression of β-Catenin (a key molecule of wnt-related metastasis pathways), but it did up-regulate the expression of two EMT marker proteins, E-Cadherin and ZO-1, and down-regulate N-Cadherin. These results indicate that Rh3 might inhibit the migration and invasion of colorectal cancer cells (HCT116) via suppression of the EMT.

## 4. Materials and Methods

### 4.1. Cell Lines and Reagents

The human colorectal cancer cell line, HCT116, and human normal colon epithelial cells, HCoEpiC, were purchased from the American Type Culture Collection (Manassas, VA) and cultured in RPMI-1640 medium (HyClone, Logan, UT, USA), with 10% (*v*/*v*) heat-inactivated fetal bovine serum (FBS, Zhejiang Tianhang Biotechnology Co., Ltd., Hangzhou, China), 100 U/mL penicillin, and 100 μg/mL streptomycin (Solarbio Life Sciences, Beijing, China). The cells were incubated at 37 °C in a humidified atmosphere with 5% CO_2_. All experiments were performed with cells in the logarithmic growth phase. Ginsenoside Rh3 was purchased from Shanghai Yuan Ye Biotechnology Co., Ltd. (Shanghai, China), and had a purity of 98% or greater. Rh3 was dissolved in dimethyl sulfoxide (DMSO, 20 mg/mL stock solution) prior to administration.

### 4.2. Cell Proliferation Assay

Cell growth inhibition was measured using the Cell Counting Kit-8 (CCK-8, Beyotime, Shanghai, China). Briefly, HCT116 or HCoEpiC cells were first seeded in a 96-well plate (4 × 10^3^ cells/well in 100 μL). After 24 h, the medium was changed, with the new medium containing different concentrations of Rh3 (25, 50, 75, 100, 125, 150 or 200 μg/mL). After another 24 h, 10 μL of the CCK-8 reagent was added into each well, and the plate was incubated for 2 to 3 h at 37 °C. Absorbance (A) at 450 nm was then measured using a microplate ELISA reader (Perlong, Beijing, China). The inhibition of cell growth was calculated as:

Inhibition = [1 − (A_sample_ − A_blank_)/(A_cell control_ − A_blank_)] × 100% (1)

The half-maximal inhibitory concentration (IC_50_) was calculated using the R package IC_50._ All of these experiments were performed in triplicate.

### 4.3. Selectivity Index (SI)

The degree of selectivity of Rh3 was calculated as previously reported [44] with minor modification:

SI_Rh3_ = (IC_50_ in HCoEpiC cells)/(IC_50_ in HCT116 cells) (2)

### 4.4. Cell Migration and Invasion Assay

Transwell migration and invasion assays were performed using established procedures with a 24-well transwell plate with 8.0-µm pores (Corning Life Sciences, Corning, NY, USA), except that the upper chambers were coated with about 100 μL of Matrigel (BD Biosciences, Franklin Lakes, NJ, USA) for the invasion assay. HCT116 cells were suspended in serum-free medium containing 80 μg/mL of Rh3 (or an equal amount of DMSO) and seeded into the upper chambers of the transwell plate (4 × 10^5^ cells/well in 350 µL). Then, 700 µL of chemoattractant medium with 15% FBS was added into the lower chamber. After incubation for 48 h, cells that invaded through the membrane were fixed with paraformaldehyde, stained with crystal violet (Beyotime), and observed under a microscope at 100× (Nikon E100, Nikon Corp, Tokyo, Japan). Cell invasion was counted in five randomly selected fields.

### 4.5. Cell Apoptosis and Cell Cycle Analyses

Cell apoptosis and cell cycle progression were determined using the Annexin V and propidium iodide (PI) kit (Beyotime, China). Briefly, HCT116 cells were seeded into a 6-well plate (1 × 10^6^ cells/well in 3 mL), and 80 μg/mL Rh3 (or an equal amount of DMSO) was added the next day. For determination of apoptosis, cells were harvested after 24 h of Rh3 treatment, washed twice with PBS, and suspended in 500 μL of binding buffer. Then, 5 μL of Annexin V-FITC was added for 15 min and 3 μL of PI was added for 5 min in the dark at room temperature. The cells were collected by centrifugation and resuspended in 400 μL of binding buffer for flow cytometry.

For cell cycle analysis, cells were harvested after Rh3 treatment for 24 h, washed with PBS, fixed with ice-cold 70% ethanol, and then incubated at 4 °C overnight. The cells were collected by centrifugation, washed with PBS, treated with 500 μL of PI staining solution and then with RNase for 30 min at 37 °C in darkness. Standard flow cytometry procedures (BD FACSCalibur Flow Cytometer, BD Biosciences) were used to analyze the cell cycle distribution. The percentage of cells in each phase was calculated by software ModFit LT.

### 4.6. RNA-Seq

HCT116 cells were seeded into a 6-well plate (1 × 10^6^ cells/well). After 24 h, the original medium was discarded, and a medium with Rh3 (80 μg/mL) was added for another 24 h. Then, this medium was discarded; the cells were washed with PBS, lysed, and then preserved with Trizol. RNA purification, library preparation, and RNA-sequencing were performed by GENEWIZ Co. (Beijing, China). Total RNA was extracted with Trizol reagent, libraries were constructed using NEBNext^®^ UltraTM RNA Library Prep Kit for Illumina^®^ (NEB, Ipswich, MA, USA), and library preparations were sequenced on the Illumina Novaseq platform.

### 4.7. Analysis of RNA-Seq Data

Most of the data in this study were analyzed using R software and selected packages (http://www.r-project.org, accessed on 9 December 2020). The quality of reads was assessed by FastQC. The adapter was removed by Cutadapt (version 1.9.1), and mapped onto the Ensembl human reference genome (EnsemblGRCh37 release 98) using the aligner software Hisat2 (v2.0.1) [45]. The DEGs were analyzed using the R package EdgeR [46] and a heatmap was constructed using the R package pheatmap [47]. GO and KEGG GSEA were performed and visualized using GSEA version 4.1.0 (http://www.gsea-msigdb.org/gsea/index.jsp, accessed on 5 January 2021).

### 4.8. Colon Cancer Data Acquisition and Analysis

Data on the mRNA expression profiles of colon cancers were downloaded from The Cancer Genome Atlas (TCGA) website (https://portal.gdc.cancer.gov/, accessed on 21 December 2020). These data included 648 samples from colon cancer patients (482 samples from tumor tissues and 166 samples from adjacent normal tissues) and clinical information about each patient. Log_2_-fold change (log_2_FC) of gene expression (with a focus on genes related to DNA replication) between tumor and normal tissues were analyzed using the R package EdgeR [46].

### 4.9. Western Blotting and Co-IP

For Western blotting experiments, HCT116 cells were seeded into a 6-well plate (1 × 10^6^ cells/well in 3 mL), and 80 μg/mL Rh3 (or DMSO) was added the next day. After 24 h, total protein was extracted using a RIPA lysis buffer (P0013D, Beyotime). Proteins were then separated using 10% SDS-PAGE and transferred to a PVDF membrane (Thermo Fisher Scientific, Shanghai, China). Membranes were immersed in 5% nonfat milk or 5% bovine serum albumin (BSA), and then incubated with primary antibodies from Cell Signaling Technology (CST) or Beyotime: Mcm2 (3619T, 1:2000, CST), phosphorylated Mcm2 (8861S, 1:3000, CST), Cdt1 (8064S, 1:3000, CST), Orc6 (4737S, 1:2000, CST), β-Catenin (8480S, 1:2000, CST), MMP-9 (13667S, 1:1000, CST), LOX (58135S, 1:1000, CST), ZO-1 (13663S, 1:2000, CST), E-Cadherin (3195S, 1:1000, CST), N-Cadherin (13116S, 1:1000, CST), Vimentin (5741S, 1:1000, CST) and β-actin (AF0003, 1:2500, Beyotime). A goat anti-rabbit IgG H + L (HRP-labeled; A0216, 1:10,000, Beyotime) and a goat anti-mouse IgG H + L (HRP-labeled; A0208, 1:10,000, Beyotime) were then added, and the signals were detected using ECL reagent (Beyotime).

For Co-IP experiments, total proteins were extracted using a RIPA lysis buffer. Then the Orc6 antibody was added, and the samples were incubated at 4 °C overnight. Then, protein G magnetic beads (MedChemExpress, Monmouth Junction, NJ, USA) were added for 1 h at 4 °C to isolate the Orc6 antibody and bound proteins. The beads were washed five times with lysis buffer to remove as much of the wash buffer as possible, and the beads were then suspended in 30 µL of 1 × SDS loading buffer and boiled for 5 min. All subsequent steps were the same as those for Western blotting.

## 5. Conclusions

The present study showed that ginsenoside Rh3 inhibited the proliferation, invasion, and migration of HCT116 cells, and also led to cell cycle arrest and increased apoptosis. GSEA of changes in the transcriptome profile indicated that the greatest effect of Rh3 was on DNA replication. Most notably, the same DNA replication genes that were down regulated by Rh3 in our HCT116 experiments were up regulated in colon cancer tissues from TCGA. Our results, therefore, suggest that Rh3 treatment should be considered in future in vivo studies of colon cancer.

## Data Availability

The raw data of RNA-seq were submitted to the Sequence Read Archive (SAR) of National Center for Biotechnology Information. BioProject ID PRJNA765451. https://www.ncbi.nlm.nih.gov/bioproject/PRJNA765451 (accessed on 3 August 2022).

## Supplementary Materials

The following supporting information can be downloaded at https://www.mdpi.com/article/10.3390/molecules27155002/s1. Figure S1: Effect of Rh3 (80 μg/mL) on morphology of HCoEpiC cells. Figure S2: Heatmap of expression of random selected genes. Table S1: GSEA_GO. Table S2: GSEA_KEGG.

## Sample Availability

Samples of the compounds are available from the authors.
