# Supplementary figures and images for: Transcriptome Analysis of the Anti-Proliferative Effects of Ginsenoside Rh3 on HCT116 Colorectal Cancer Cells
Source: Molecules. 2022 Aug 6;27(15):5002. doi: 10.3390/molecules27155002 (PMC9370307; doi:10.3390/molecules27155002)

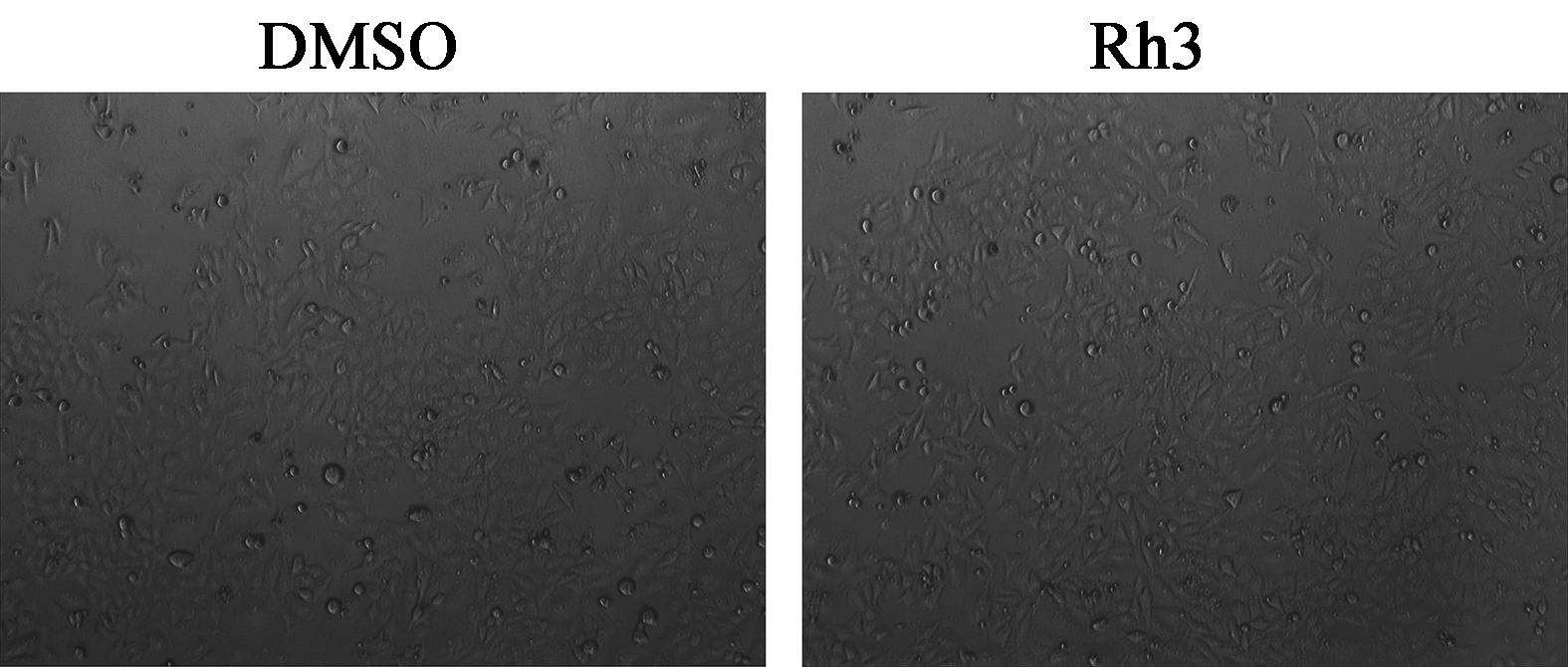

Supplement: Supplementary file 1 [file molecules-27-05002-s001.zip › Figure S1 Effect of Rh3 (80 a╠g_mL) on morphology of HCoEpiC cells.tif]

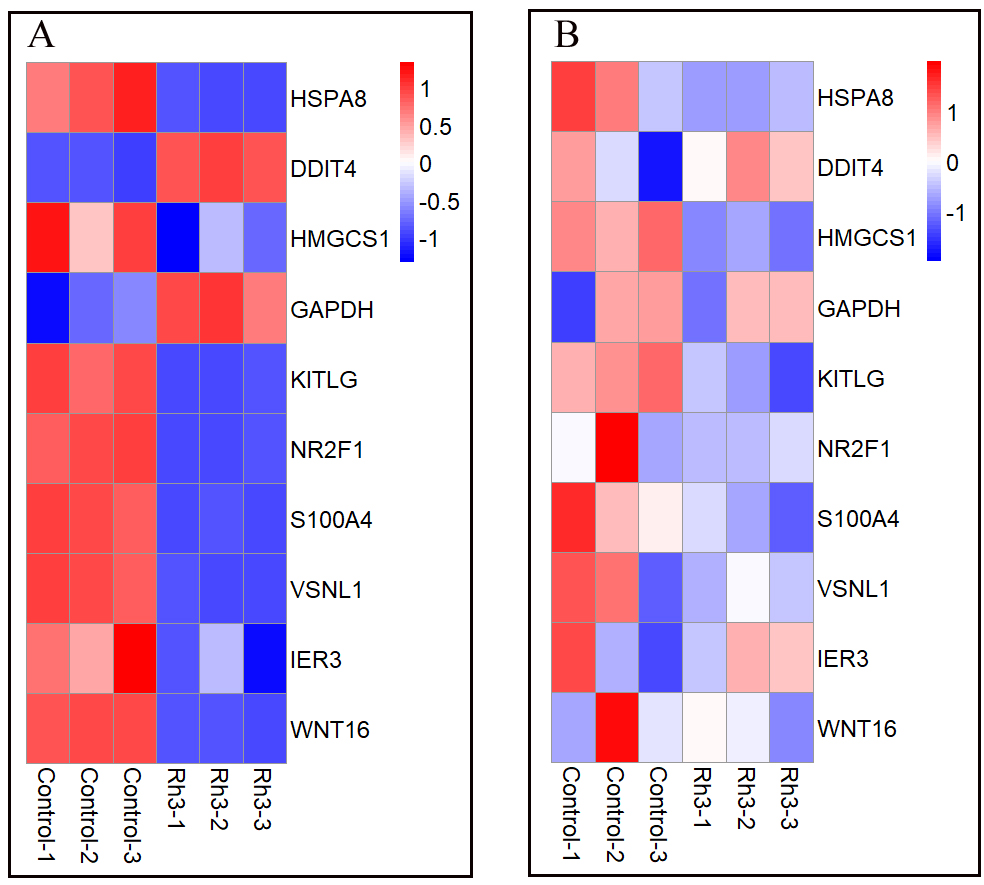

Supplement: Supplementary file 1 [file molecules-27-05002-s001.zip › Figure S2 Heatmap of expression of random selected genes (A-RNA-seq and B-qPCR).jpg]
